# A Distinct Slow-Cycling Cancer Stem-like Subpopulation of Pancreatic Adenocarcinoma Cells is maintained *in Vivo*

**DOI:** 10.3390/cancers2042011

**Published:** 2010-11-29

**Authors:** Jennifer L. Dembinski, Stefan Krauss

**Affiliations:** Cellular and Genetic Therapy, Department of Microbiology, Cancer Stem Cell Innovation Center (CAST), Oslo University Hospital, Rikshospitalet, Oslo, Norway

**Keywords:** cancer stem cell, slow-cycling cells, pancreas adenocarcinoma, EMT

## Abstract

Pancreatic adenocarcinoma has the worst prognosis of any major malignancy, with <5% of patients surviving five years. This can be contributed to the often late diagnosis, lack of sufficient treatment and metastatic spread. Heterogeneity within tumors is increasingly becoming a focus in cancer research, as novel therapies are required to target the most aggressive subpopulations of cells that are frequently termed cancer stem cells (CSCs). In the current study, we describe the identification of a slow-cycling cancer stem-like population of cells *in vivo* in BxPC-3 and Panc03.27 xenografts. A distinct slow-cycling label-retaining population of cells (DiI+/SCC) was found both at the edge of tumors, and in small circumscribed areas within the tumors. DiI+/SCC in these areas display an epithelial-to-mesenchymal transition (EMT) fingerprint, including an upregulation of the mesenchymal markers vimentin and N-cadherin and a loss of the epithelial marker E-cadherin. DiI+/SCC also displayed a critical re-localization of beta-catenin from the membrane to the nucleus. Additionally, the DiI+/SCC population was found to express the developmental signaling molecule sonic hedgehog. This study represents a novel step in defining the biological activities of a tumorigenic subpopulation within the heterogeneous tumor microenvironment *in vivo*. Understanding the interactions and functions of a CSC population within the context of the tumor microenvironment is critical to design targeted therapeutics.

## 1. Introduction

Pancreatic adenocarcinoma has the worst prognosis of any major malignancy, and although progress has been made in the last two decades, the advancements have not yielded much improvement in the outcome of the disease [1]. More recently, central molecular pathways that are involved in pancreatic adenocarcinoma have been elucidated [2], and parameters that indicate heterogeneity in the tumor *in vitro* have been described, including cancer stem cells (CSCs), with increased chemotherapy resistance and mobility [3,4]. The occurrence and unique properties of CSCs in pancreas adenocarcinoma justify focusing on these cells for further understanding and possibly improving the treatment of this disease.

To date, CSCs have been identified in a variety of solid tumors including; breast cancer [5], colon carcinoma [6], melanoma [7], pancreatic adenocarcinoma [4,8,9] and prostate cancer [10] using various methods of detection. While CSC populations share similar characteristics regardless of tumor type, including the capacity for self-renewal, high tumor initiating potential and relative quiescence, they have been isolated and characterized using numerous techniques. Methods that have been used to identify CSCs include cell surface markers such as CD20, CD24, CD44, CD133, epithelial-specific antigen (ESA), aldehyde dehydrogenase activity (ALDH), efflux activity (side-population cells), and most recently, label-retention [3,4,5,6,7,8,9,11,12,13,9,11].

CSC regulation has not yet been well defined, but several developmental signaling pathways such as hedgehog and Wnt that are implicated in the self-renewal process of normal stem cells, have been identified in CSCs [14,15]. Human pancreatic adenocarcinomas display increased hedgehog pathway activity, and overexpression of sonic hedgehog (Shh) within the pancreas results in the development of PanIN lesions; a precursor to pancreatic cancer [16]. Specifically, it has been determined that CSCs themselves are mainly responsible for Shh expression as they have been found to significantly overexpress the ligand while persistent, albeit lower, amounts are expressed by the bulk tumor cells [9]. Hedgehog-Gli1 signaling has also been implicated in epithelial-to-mesenchymal transition (EMT), and increases in the levels of pathway components (particularly Gli1) parallels the progression of carcinoma stem cells to metastatic states [17]. Gli1 induces the transcription of Snail, a zinc-finger protein that represses transcription of E-cadherin to promote EMT. The loss of E-cadherin leads to a relocation of beta-catenin from the cell membrane to the nucleus, thus allowing the Shh–Gli1 pathway to participate in the mechanisms that induce beta-catenin relocation to the nucleus [18]. Beta-catenin, which is an integral component in the canonical Wnt signaling pathway, is also implicated in CSC regulation. While there is controversy in what the presence or role of active Wnt signaling in CSCs is [19], nuclear accumulation of beta-catenin seems to consistently be implicated in enhanced metastatic potential and poor prognosis in various solid tumors [20,21]. In addition, a variety of further pathways have been implicated in modulating beta-catenin accumulation in the nucleus, including HGF [22] and PDGF [23]. Importantly, these factors can be regulated by the microenvironment. For example, stromal fibroblasts or tumor associated fibroblasts (TAFs) modulate the environment by secreting growth factors (such as HGF and PDGF) [21,24,25,26], and therefore the tumor microenvironment may drive tumor growth and even selectively support a subset of tumor cells, such as CSCs. [21].

Previous reports found that nuclear beta-catenin positive cells are distributed non-randomly throughout a tumor. In addition, it has been reported that cells with nuclear beta-catenin tend to localize at the edge or invasive front of the tumor [21,27] where most invasive cells are typically located. Multiple reports confirm the presence of typical EMT markers such as expression of vimentin, loss of E-cadherin, and nuclear beta-catenin accumulation at the invasive edge of a tumor [21,26,27]. Clinically, it is important to correlate molecular biomarkers such as those involved in EMT with patient treatment outcome [28].

Interestingly, although one would suspect that cells with increased migratory properties share characteristics of CSCs, such strict correlation has not been confirmed to date. However, a recent article by Ganepola *et al*. has reported that metastatic lesions have a lower proliferative rate compared to primary tumors, suggesting that the reduced proliferation rate in metastatic tumors is either a consequence of tumor cells with slower cycling speeds being more adapt to embed and propagate within a new environment or that it reflects altered growth signals within the new environment [29].

We have previously identified a slow-cycling, label-retaining cancer stem-like subpopulation of cells in pancreatic adenocarcinoma cell lines which has undergone EMT and is functionally more invasive and tumorigenic [3]. In this report, we further characterize this population of cells and describe the interactions of this cancer stem-like population within the tumor microenvironment.

Our findings represent a further step in understanding the role of CSCs in tumor progression and thus, may help in the future development of novel therapeutics that can target this critical subpopulation.

## 2. Results and Discussion

### 2.1. In Vivo DiI+/SCC Identification

**Figure 1:**
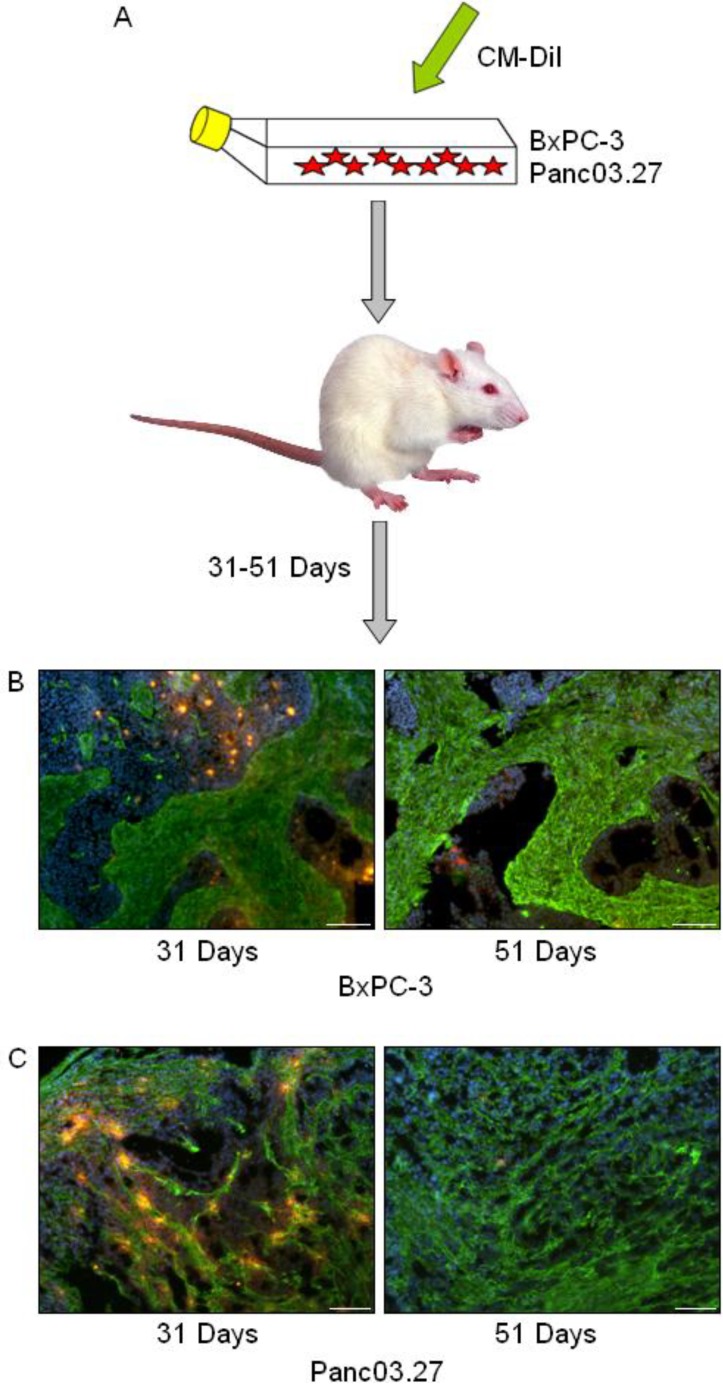
*In vivo* DiI+/SCC Selection. (**A**) Schematic diagram: BxPC-3 or Panc03.27 cells were labeled with CM-DiI and subcutaneous tumors were established in CB17/SCID mice. Tumors were harvested after 31 or 51 days and processed for immunohistochemical analysis. (**B**) BxPC-3 and (**C**) Panc03.27 tumor sections from 31 or 51 day tumors displaying a reduction in DiI+ cells (red) over time. Slides are additionally stained against fibroblasts (Alexa Fluor 488, green) to show stromal networks and counterstained with Dapi (blue). Bars represent 100 µm.

In a previous report we have identified the occurrence of slow cycling cells (DiI+/SCC) with tumor stem cell-like properties in cell cultures of established pancreas adenocarcinoma cell lines [3]. To assess whether such cells may also be present in the context of a tumor microenvironment, tumors were established using freshly labeled DiI+ BxPC-3 or Panc03.27 cells, harvested at early (31 days) and late (51 days) time points, and processed for further immunohistochemical analysis (Figure 1A). To establish whether DiI+/SCC identification was possible *in vivo* (and to determine the time frame), a pilot study was carried out where subcutaneous tumors were established in mice using DiI labeled cells and harvested after 22, 33, 35, 44, 46 or 54 days. DiI intensity was subsequently evaluated in tumor sections and it was noted that while the number of DiI+ cells decreased with time, even in our furthest time point (54 days) DiI+ cells still remained. We therefore decided to use the longer time point for our full scale experiment. Examples of tumors harvested at the early (31 days) and late (51 days) time points can be seen in Figure 1B (BxPC-3) and 1C (Panc03.27), where slides were stained against fibroblasts (Alexa Fluor 488, green) and nuclei were counterstained with Dapi (blue) to highlight the epithelial and stromal tumor compartments. BxPC-3 tumors harvested at 31 days had on average 35% DiI+ epithelial tumor cells while at 51 days the DiI+ population was reduced to 5%, which is roughly the percentage of DiI+/SCC we obtained by FACS analysis when characterizing this population after six weeks growth *in vitro*. Similarly, in the Panc03.27 xenografts, 49% of the epithelial tumor cells remained DiI+ after 31 days, while at 51 days 3% of the cells retained DiI+, which again reflected approximately the percent of DiI+/SCC we acquired by FACS after four weeks of growth *in vitro* [3]. Subsequent results thus refer to the 51 day tumor time point.

### 2.2. DiI+/SCC Form a Distinct Subpopulation *In Vivo*

**Figure 2:**
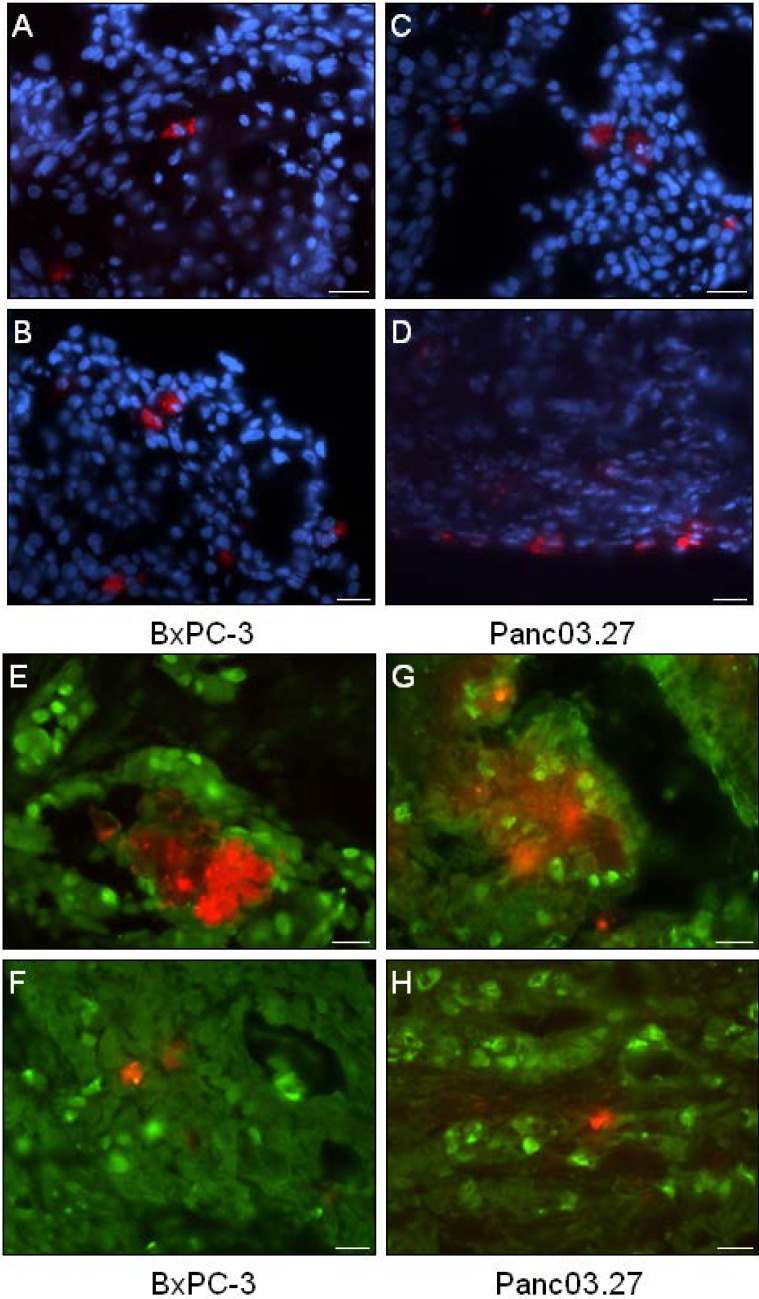
Distribution and Proliferation of DiI+ Cells. (**A**-**D**) Tumor sections were stained with Dapi (blue) to evaluate the location of DiI+ cells (red). (**A**-**B**) BxPC-3 tumor sections and (**C**-**D**) Panc03.27 tumor sections. (**A** and **C**) single DiI+ cells within the tumor and (**B** and **D**) on the tumor edges. (**E**-**H**) Immunohistochemical analysis of the cellular proliferation marker Ki67 (Alexa Fluor 488, green). (**E**-**F**) BxPC-3 and (**G**-**H**) Panc03.27 tumor sections. DiI+ cells (red) were confirmed quiescent (Ki67-) whether situated in clusters (**E** and **G**) or as single cells (**F** and **H**). Bars represent 20 µm.

Next, the distribution of DiI+ cells was analyzed. As seen in Figure 2(A-D), DiI+ cells were mainly seen as single cells, however occasionally small clusters of DiI+ could be found (Figure 2E and G). Single DiI+ cells could be found both within the tumor (Figure 2A and C) as well as on the tumor edges (Figure 2B and D).

To verify that the xenografted DiI+ label-retaining cells reflected the properties of DiI+/SCC as determined *in vitro*, and were in a quiescent state, tumor sections were stained against the cellular proliferation marker Ki67 (Figure 2E-H). Positive Ki67 staining was detected in the nuclei of DiI negative human epithelial tumor cells (DiI-/FCC), while both clustered DiI+ cells (Figure 2E and H) and single DiI+ cells (Figure 2F and H) were found to be Ki67-negative (quiescent), suggesting the label retaining DiI+ cells were in fact DiI+/SCC.

**Figure 3:**
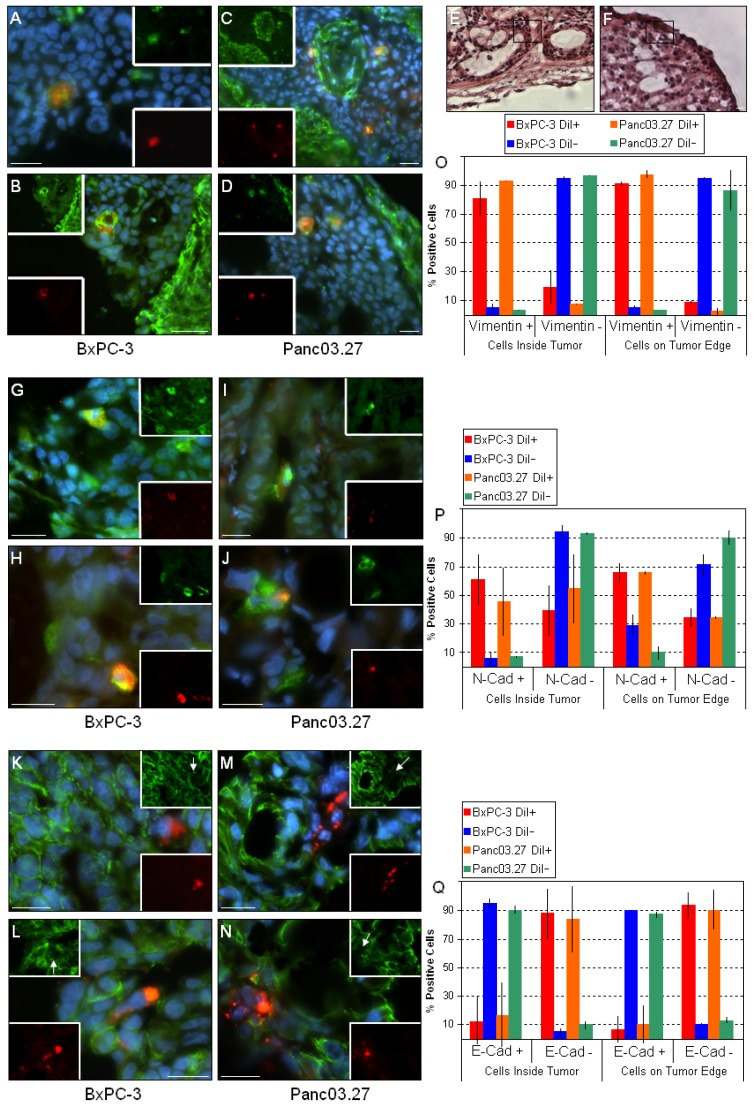
DiI+/SCC undergo EMT *in vivo.* Sections from BxPC-3 and Panc03.27 tumors were stained against the mesenchymal markers vimentin (**A**-**D**) and N-cadherin (**G**-**J**) and the epithelial marker E-cadherin (**K**-**N**) (Alexa Fluor 488, green). DiI+/SCC (red) located within the tumor (**A** and **C**; **G** and **I**; **K** and **M**) or on the tumor edge (**B** and **D**; **H** and **J**; **L** and **N**) stained positive for the mesenchymal markers and negative for epithelial markers, suggesting an EMT. (**E** and **F**) corresponding H&E sections for the vimentin stained sections **B** and **D**, respectively. The boxed region represents the area displayed in **B** and **D**, confirming their location on the tumor edge. (**O**) Graphical representation of statistical analysis for vimentin. Nearly all DiI+/SCC were vimentin +, while DiI-/FCC were mainly vimentin -. (**P**) Graphical representation of statistical analysis for N-cadherin. More than half of the DiI+/SCC were N-cadherin +, while most of the DiI-/FCC remained N-cadherin -. (**Q**) Graphical representation of statistical analysis for E-cadherin. Almost all DiI+/SCC were E-cadherin – while DiI-/FCC remained E-cadherin +. Bars represent 20 µm.

Next, the distribution of the mesenchymal markers vimentin (Figure 3A-D) and N-cadherin (Figure 3G-J), and the epithelial marker E-cadherin (Figure 3K-N) were assessed. As expected, the mesenchymal stromal tumor compartments stained positive for both mesenchymal markers [24,27,30]. When the epithelial tumor compartments were examined, it was found that nearly all DiI+/SCC were vimentin positive in both tumor types, regardless whether located within the tumor (Figure 3A and C) or on the tumor edges (Figure 3B and D). Similarly, most DiI+/SCC also stained positive for N-cadherin within the tumors (Figure 3G and I) and on the tumor edges (Figure 3H and J). Correspondingly, when E-cadherin staining was assessed, virtually all DiI+/SCC were E-cadherin negative regardless of their location (within the tumor: Figure 3K and M; tumor edges: Figure 3L and N), while almost all other cells in the epithelial tumor compartments stained E-cadherin positive. Additionally, as shown in Figure 3O, P and Q, we noted a slight increase in the mesenchymal markers and decrease in the epithelial markers in the DiI+/SCC which were located on the tumor edge. This suggests that while the bulk of cells in the epithelial tumor compartments (*i.e.*, DiI-/FCC) are in fact of an epithelial phenotype, the DiI+/SCC express mesenchymal markers and have likely undergone EMT. This is consistent with our previous findings, where DiI+/SCC showed a clear molecular fingerprint of an EMT combined with a functional increased invasiveness *in vitro* as determined by a matrigel invasion assay [3]. In particular, the upregulation of vimentin and N-cadherin and loss of E-cadherin at the invasive front of tumors has been linked to metastasis and poor clinical outcome [27,30]

**Figure 4:**
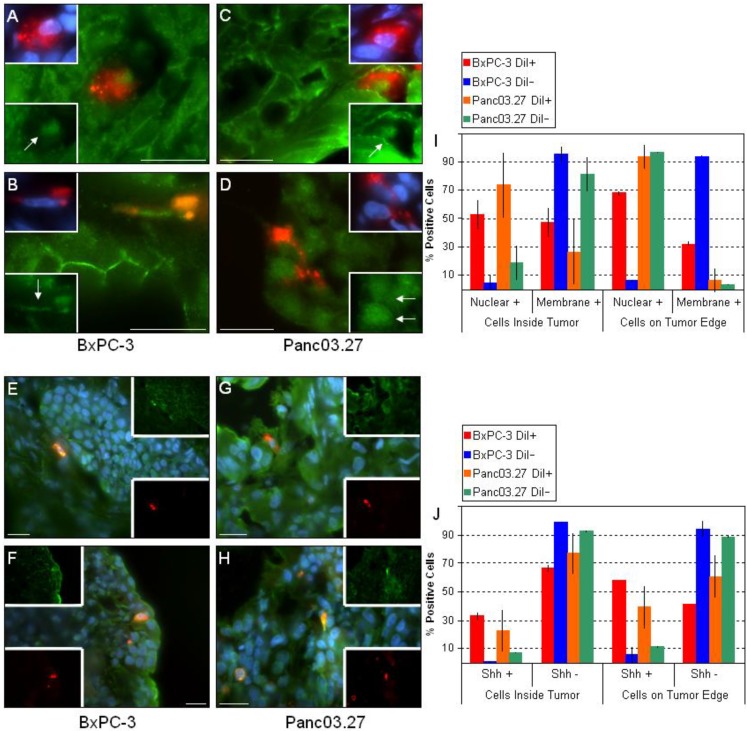
Key CSC Signaling Pathways. Sections from BxPC-3 and Panc03.27 tumors were stained against active beta-catenin (**A**-**D**) or Shh (**E**-**H**). DiI+/SCC tended to have nuclear + beta-catenin within the tumor (**A** and **C**) and increased nuclear beta-catenin on the tumor edge (**B** and **D**). (**I**) Graphical representation displaying nuclear *vs.* membrane staining of beta-catenin in DiI+/- cells inside the tumor or on the tumor egdes. Similarly, DiI+/SCC stained + for Shh as opposed to DiI-/FCC (**E**-**H**). DiI+/SCC increased expression of Shh when on the tumor edge (**F** and **H**) as compared to within the tumor (**E** and **G**). (**J**) Graph displaying percent Shh positive DiI+/- cells and location within the tumor. Bars represent 20 µm.

Next, we looked at the key pathways implicated in stem cell regulation: Wnt and Shh. Numerous sources have associated aberrant Wnt and/or Shh signaling with CSCs [4,9,20,26,31] and we have previously determined *in vitro* that the DiI+/SCC population had an up to four-fold increase in gene expression of Shh and parts of its pathway components as compared to the DiI-/FCC population, and that the expression of selected Wnts was elevated (Wnt5a and Wnt10b) [3]. Additionally, we observed an increase in nuclear beta-catenin in the DiI+/SCC population *in vitro* by ICC/IF (data not shown). To assess the activity of these critical pathways within the tumor microenvironment, we stained the tumor sections against active beta-catenin (Figure 4A-D) and Shh (Figure 4E-H). Interestingly, nearly all of the DiI-/FCC in the epithelial tumor compartments located inside the tumors (Figure 4A and C) showed positive membrane/cytosol beta-catenin staining, while most of DiI+/SCC had positive nuclear beta-catenin staining. Thus, in the bulk of the epithelial tumor cells (DiI-/FCC) ,beta-catenin remains membrane bound, and may be linked to E-cadherin, while in the mesenchymal DiI+/SCC, beta-catenin is translocated to the nucleus [18]. Additionally, when the tumor edges were assessed, we found that the percent of nuclear positive DiI+/SCC increased.

In line with recently published data [20,27], these data suggest that DiI+/SCC tend to display a more malignant phenotype compared to bulk cells and those that are located on the tumor edge have a greater likelihood of taking on an invasive malignant phenotype. One cannot dismiss that single DiI+/SCC within the tumor may be actively migrating (‘migrating cancer stem cell’(MCSC)) enroute to the invasive tumor front [20].

The observed molecular profile of DiI+/SCC in xenografts, including an upregulation of mesenchymal markers such as vimentin, the loss of E-cadherin expression and accumulation of nuclear beta-catenin, are poor prognostic markers that are correlated with the conversion of early-stage tumors to invasive malignancies [27]. As such, different levels of nuclear beta-catenin are likely to reflect altered malignant behavior [20,32].

What may drive the occurrence of DiI+/SCCs? In the case of the pancreas adenocarcinoma cell lines analyzed here, the *in vitro* experiments point towards an autocrine mechanism. However, the tumor microenvironment itself may also drive growth of a particular subset of cells, such as CSCs, and contribute to malignancy. For example tumor associated fibroblasts have been shown to secrete a variety of factors (such as hepatocyte growth factor (HGF)) that have been reported to influence nuclear beta-catenin accumulation [24,33,34,35]. Therefore, one cannot exclude the possibility that the presence of nuclear beta-catenin in DiI+/SCC may not simply be a result of active canonical Wnt signaling, but may also be triggered by a relocation of cytoplasmic/membrane fractions of beta-catenin in response to the release of growth factors, chemokines, *etc*. from the tumor microenvironment.

Pancreatic adenocarcinomas have been shown to display increased Shh pathway activity, and specifically, pancreatic CSCs themselves significantly overexpress the ligand. In a study by Li *et al*. that evaluated the level of Shh transcripts, bulk pancreatic cancer cells displayed a four-fold increase as compared to normal pancreatic epithelial cells while pancreatic CSCs showed a 46-fold increase [9]. Additionally, Gli1, the transcription factor activated by Shh, was found to induce Snail, a repressor of E-cadherin, implicating that the Shh-Gli1 pathway may contribute to the relocation of beta-catenin from the cell membrane to nucleus, and may thus assist in triggering an EMT [18]. When Shh expression was evaluated in our tumor sections, we found that Shh was preferentially expressed by the DiI+/SCC population (Figure 4E-H). In addition, as previously observed, we saw that the frequency of DiI+/SCC expressing Shh increased when comparing the inside of the tumor (Figure 4E and G) to the tumor edges (Figure 4F and H), while the frequency of positive DiI-/FCC remained the same throughout. The ratio of Shh positive DiI+ or DiI- cells and their location in the tumor can be seen in Figure 4 J.

## 3. Experimental Section

### 3.1. Cells and Culture Conditions

BxPC-3 and Panc03.27 pancreatic adenocarcinoma cells were obtained from ATCC and were cultured as described previously [3].

### 3.2. Animals

Four-to-six week old female CB17/SCID mice were used throughout this study. All mice were housed and used under the approved protocols in accordance with the National Institute of Health guide for the care and use of laboratory animals, and all efforts were made to minimize the number and suffering of animals.

### 3.3. Tumor Establishment/ In Vivo DiI+/SCC

To investigate whether *in vivo* SCC identification was possible, a pilot study was carried out where subcutaneous tumors were established in CB17/SCID mice by injecting 5 × 10^6^ DiI+ labeled BxPC-3 or Panc03.27 cells (Vybrant® DiI cell-labeling solution; (Invitrogen/Molecular Probes)) in 200 µL PBS as described previously [3]. Tumors were harvested after 22, 33, 35, 44, 46 or 54 days, embedded in Tissue TEK OCT compound (Electron Microscopy Sciences), snap-frozen and stored at –80 °C (data not shown). Frozen tissue was sectioned (6–8 µm), mounted onto slides, and evaluated via multiple immunohistochemistry/immunofluorescence (IHC/IF) assays.

For the full scale experiment, tumors (n = 6 per tumor type) were established (sc) using 5 × 10^6^ DiI+ labeled BxPC-3 or Panc03.27 cells (Vybrant® CM-DiI cell-labeling solution (Invitrogen/Molecular Probes)). Tumors were harvested after 51 days, embedded in Tissue TEK OCT compound, snap-frozen and stored at –80 °C. Frozen tissue was sectioned (6-8 µm), mounted onto slides, and used in subsequent IHC/IF analyses as described below.

### 3.4. Immunohistochemistry/immunofluorescence (IHC/IF)

Slides with sections of frozen tissue were fixed with 4% paraformaldehyde for 10 minutes, permeabilized with 0.2% Triton-X in PBS for 10 minutes (β-catenin, vimentin, N-cadherin, E-cadherin, Ki67, Fibroblasts), blocked for 30 minutes at room temperature with 3% BSA in PBS and incubated for 16 hours at 4 °C with either rabbit anti-Ki67 (Novocastra (NCL-Ki67p) 1/100 dilution), mouse anti-N-cadherin (Santa Cruz (sc-8424), 1/100 dilution), mouse anti-E-cadherin (Abcam (ab1416); 1/100 dilution), mouse anti-vimentin (Abcam (ab8978); 1/200 dilution), mouse anti-active-β-Catenin (anti-ABC), clone 8E7 (Millipore; 1/300 dilution), rat anti-Shh (Abcam (ab50515); 25µg/ml), or rat anti-fibroblasts (Santa Cruz (sc-73355), 1/200 dilution) in 1% BSA in PBS. Secondary antibodies used were Alexa Fluor 488 goat anti-mouse, Alexa Fluor 488 goat anti-rabbit, Alexa Fluor 488 donkey anti-rat (Molecular Probes; 1/700 dilution) in 1% BSA in PBS for 1 hour at RT. Nuclei were counterstained with Dapi (Roche, 1 mg/ml), in PBS for 5 minutes at RT. Alternatively, sections were fixed using cold acetone and stained with hematoxylin and eosin. Images were obtained on an Axiovert 200M microscope (Zeiss) using Axiovision software. Electronic images were further processed using Adobe Photoshop.

Statistics: DiI+ or DiI- cells with Dapi stained nuclei were counted in the epithelial tumor cell compartments of the tumor sections (n = 6) whether inside the tumor or on the tumor edge (tumor edge was defined as within one 40× (oil) field from the edge). Graphs display average with standard deviation error bars.

## 4. Conclusions

In this report, we further define a slow-cycling cancer stem-like cell subpopulation in pancreatic adenocarcinoma cell lines *in vivo* and demonstrate the characteristics of this important subpopulation in the context of the tumor microenvironment. The presence of a distinct population of tumor cells, DiI+/SCC, was confirmed to comprise a slowed cell cycle, upregulated expression of mesenchymal markers, a loss of epithelial markers, increased expression of Shh and nuclear beta-catenin accumulation.

The expression of the mesenchymal markers vimentin and N-cadherin and loss of the epithelial marker E-cadherin suggests these once epithelial tumor cells have undergone an EMT; characteristic of aggressive, invasive cells such as CSCs [27,36]. Additionally, the accumulation of beta-catenin in the nucleus has been associated with the metastatic conversion of epithelial cells and tumor invasion. The re-localization of beta-catenin from the membrane to the nucleus is directly correlated with a loss of E-cadherin expression, and is preferentially found in cells on the invasive edges of tumors [18,27]. In a study aimed at identifying pancreatic CSCs in tissue samples derived from patients, it was demonstrated that at the invading front of pancreatic tumors, CSCs, as defined by the presence of CD133+, also expressed CXCR4. While CD133+CXCR4- and CD133+CXCR4+ cells were able to form tumors, only CD133+ CXCR4+ cells were able to metastasize. This implies that there are two distinct phenotypes of CSCs; stationary and migratory forms [37]. Heterogeneity within the CSC population was also reported when CD24^+^/CD44^+^ pancreatic CSCs were analyzed against the CD133^+^ CSC population, where only 10–40% of cells were found to overlap and highly variability was seen [37].

The label-retention technique that we have previously used *in vitro* [3] appears to reliably identify and locate slow-cycling cells *in vivo*. However, the technique has also some restrictions: DiI+/SCC can be founder cells for DiI-/FCC *in vitro*, but also DiI-/FCC can develop into slow cycling DiI+/SCC [3]. It is likely that the same occurs *in vivo*, and thus a percentage of cells that behave as SCC, are not stained with DiI as they developed from DiI-/FCC. In an effort to combat this issue, we are currently in the process of characterizing live cell reporters that reliably mark the SCC population *in vitro* and *in vivo*.

The technique employed here for identifying slow-cycling cancer stem like-cell subpopulations *in vivo* can aid in the understanding of tumor heterogeneity and the development of therapeutic tools; in particular tools to target the most aggressive population of cancer cells.
